# Approach to multidrug resistant infections in pediatric transplant recipients

**DOI:** 10.3389/fped.2023.1270564

**Published:** 2023-12-07

**Authors:** Sara W. Dong, Tanvi S. Sharma, Paul K. Sue

**Affiliations:** ^1^ Division of Infectious Diseases, Department of Pediatrics, Children’s Healthcare of Atlanta and Emory University School of Medicine, Atlanta, GA, United States; ^2^ Division of Infectious Diseases, Department of Pediatrics, Boston Children’s Hospital, Boston, MA, United States; ^3^ Division of Infectious Diseases, Department of Pediatrics, Columbia University Irving Medical Center, New York, NY, United States

**Keywords:** multidrug-resistant, ESBL, CRE, transplant, pediatric

## Abstract

**Introduction:**

There is increasing recognition of infections due to multidrug-resistant Gram negative (MDRGN) bacterial infections among children undergoing solid organ and hematopoietic cell transplantation, which may be associated with morbidity and mortality.

**Methods:**

We present two vignettes that highlight the clinical challenges of evaluation, management, and prevention of MDRGN bacterial infections in children prior to and after transplantation. The goal of this discussion is to provide a framework to help develop an approach to evaluation and management of these infections.

**Results:**

Source control remains the utmost priority in management of MDR infections and is paired with antibiotic selection guided by *in vitro* susceptibilities, adverse effect profiles, and clinical response. Identification and confirmation of resistance can be challenging and often requires additional testing for recognition of complex mechanisms. Current antimicrobial approaches to MDRGN infections include use of novel agents, prolonged infusion, and/or combination therapy. We also discuss preventative efforts including infection control, antimicrobial stewardship, targeted pre-emptive or prophylactic treatment, and decolonization.

**Discussion:**

The impact of MDRGN infections on patient and graft survival highlights the need to optimize treatment and prevention strategies.

## 1. Introduction

Bacterial infections are often life-threatening in pediatric patients undergoing solid organ (SOT) or hematopoietic cell transplantation (HCT). Rapid identification and effective management of infections is essential to ensure patients safely undergo transplantation and recover from infections during vulnerable periods of associated immunosuppression. Multidrug-resistant (MDR) bacterial infections are an emerging issue in children and associated with higher mortality rates (1–4). Infections with carbapenem-resistant *Enterobacterales* (CRE) are of particular concern and especially difficult to treat. Management of MDR infections in children poses many challenges, including the need for extended susceptibility testing, which may lead to a delay in identifying effective therapeutic options, the use of novel antimicrobial agents, and the potential need for combination therapy. Furthermore, many antimicrobials with activity against MDR infections have not been well-studied in children, resulting in limited pharmacokinetics data and uncertain dosing strategies. In addition, the treatment of MDR infections among pediatric SOT and HSCT candidates is additionally complicated by immunosuppression, drug interactions, and challenges in achieving source control.

In this review, we present two clinical vignettes describing MDR Gram-negative (MDRGN) bacterial infections in children undergoing transplantation. We highlight unique considerations regarding evaluation and management of these infections, focusing on the epidemiology of infections most commonly encountered in pediatric transplant recipients, including extended-spectrum β-lactamase (ESBL)-producing *Enterobacterales* and carbapenem-resistant organisms including *Enterobacterales* and *Pseudomonas aeruginosa*, as well as antimicrobial strategies and interventions to optimize outcomes. The goal of this discussion is to present a framework that can enables infectious diseases (ID) trainees and specialists caring for the complex immunocompromised pediatric patient to develop a practical approach to diagnosis and treatment of MDRGN infections.

## 2. Clinical cases

### 2.1. Case 1

A 14-year-old male with a history of congenital hepatic fibrosis with severe portal hypertension and recurrent cholangitis presented with a 1 day history of fever, shoulder pain and acute right upper quadrant (RUQ) tenderness. His history was remarkable for recurrent episodes of cholangitis over the prior 2 years, leading to frequent courses of parenteral antibiotics as well as interval biliary stent placement and subsequent cholecystectomy. He was on longstanding fluoroquinolone prophylaxis, and at the time of admission had a marked leukocytosis of 23,000 cells/ml, elevated C-reactive protein of 19.3 mg/dl and an elevated total bilirubin of 3.1 mg/dl. Alanine transferase and aspartame transferase were within normal limits. A computerized tomography (CT) scan of the abdomen/pelvis demonstrated hepatomegaly with multiple cystic regions within the liver, consistent with dilated biliary ducts/bile lakes and the presence of pneumobilia. Piperacillin-tazobactam was started empirically, but antibiotics were escalated to meropenem due to clinical worsening. Blood culture returned positive for extended spectrum β-lactamase (ESBL) producing *Escherichia coli*, resistant to ceftriaxone, cefepime, ciprofloxacin, gentamicin, and trimethoprim-sulfamethoxazole (TMP-SMX)—but susceptible to meropenem (minimal inhibitory concentration, MIC, 0.25 μg/ml) and ertapenem (MIC 0.25 μg/ml). He received 10 days of meropenem for bacteremia and cholangitis, with rapid interval clinical resolution and was discharged home.

He required re-admission 1 week after completion of antibiotics due to fever, RUQ pain and shoulder pain, consistent with cholangitis. A repeat CT scan demonstrated persistence of biliary lakes, and cultures from a biliary drain placed into the largest collection demonstrated *E. coli*, now with resistance to meropenem (MIC 4) as well fluoroquinolones. He received a prolonged 4-week course of ceftazidime-avibactam with subsequent clinical resolution, but symptoms of biliary drainage, fever, and right sided abdominal pain recurred within 1 week of completion of antibiotics. He was listed for liver transplant as treatment for recurrent cholangitis.

### 2.2. Case 2

A 13-month-old female with a history of relapsed acute myeloblastic leukemia (AML) developed new onset left labial erythema, ulceration and fever, 6 days following allogeneic HCT. She had no prior history of bacterial infection and was on voriconazole, levofloxacin, and pentamidine prophylaxis. On workup, she was profoundly neutropenic (absolute neutrophil count of 20 cells/µl) and was started on vancomycin and piperacillin-tazobactam for febrile neutropenia with concern for labial cellulitis. Fevers continued, and she developed progressive erythema extending to the contralateral labia, multiple ulcerations to the perirectal region, and worsening hypotension on day 9 of illness. Piperacillin-tazobactam was discontinued and meropenem started. A MRI of the abdomen/pelvis demonstrated diffuse severe cellulitis of the perineum with bilateral myositis consistent with necrotizing fasciitis. She was taken to the operating room for fasciotomy, and tissue cultures demonstrated carbapenem-resistant *Pseudomonas aeruginosa*, also resistant to piperacillin/tazobactam, meropenem, imipenem, ceftazidime, aztreonam, cefepime, and fluoroquinolones but susceptible to amikacin and tobramycin.

Extended antimicrobial susceptibility testing (AST) results later demonstrated a high MIC to ceftazidime-avibactam (MIC 16/4, No Interpretation), but susceptibility to colistin (MIC 1) and ceftolozane-tazobactam (MIC 4). She subsequently showed evidence of clinical improvement with neutrophil recovery on day 11 but ultimately required two additional debridement procedures on days 12 and 14 for control of her infection.

## 3. Epidemiology of multidrug resistant gram-negative infections in the pediatric population

Antibiotic resistance can develop via multiple different mechanisms, including inactivation or alteration of the antibiotic molecule, bacterial target site modifications, reduced antibiotic penetration/accumulation, and/or the formation of biofilm (5). For the purpose of this review, the previously published international consensus definition of MDR is utilized, which is defined as nonsusceptibility to at least one agent in three or more antibiotic classes (6). A common resistance mechanism utilized by Gram negative pathogens includes enzymes that inactivate broad-spectrum β-lactam antimicrobial drugs, such as penicillins, cephalosporins, and carbapenems, via hydrolytic cleavage of the β-lactam ring. A wide variety of these unique β-lactamase enzymes exists, and several clinically important β-lactamases have emerged in both the general and transplant populations, including AmpC β-lactamases, extended spectrum β-lactamases (ESBL), and carbapenemases, which are described in further detail in Table 1.

**Table 1:** Overview of important multidrug resistance mechanisms in gram-negative pathogens (7).

Enzyme	Ambler classification^a^	Description	Mechanism/transmission	Notable organisms	Diagnosis	Geographic distribution (8)
AmpC β-lactamases	Class C (serine)	Enzymes produced at basal levels by a number of *Enterobacterales* and glucose non-fermenting Gram-negative organisms capable of hydrolyzing a number of β-lactam agents. Primary function is to assist with cell wall recycling Can be in settings of basal AmpC production or in setting of increased AmpC production	Generally occurs by one of three mechanisms - Inducible chromosomal gene expression - Stable chromosomal gene de-repression - Constitutively expressed *ampC* genes (frequently carried on plasmids but can be integrated into chromosome)	Organisms with moderate to high risk for clinically significant AmpC production due to inducible *ampC* gene include *Enterobacter cloacae* complex, *Klebsiella aerogenes*, *Citrobacter freundii* Plasmid-mediated AmpCs: *Klebsiella pneumoniae*, *Escherichia coli*, *Salmonella enteritidis*	Testing for AmpC expression is limited to research setting Cefoxitin resistance has been used to as a marker for AmpC production	Worldwide
Extended-spectrum β-lactamases (ESBL)	Class A (serine)	Heterogenous family of enzymes conferring resistance to most extended spectrum penicillins, first through third generation cephalosporins, and aztreonam Maintains *in vitro* susceptibility to cephamycins, carbapenems, β-lactamase inhibitors CTX-M enzymes are the most common ESBL encountered worldwide. Other types include TEM, SHV	Plasmids encoding ESBLs allow for efficient horizontal transmission between bacterial species Can harbor additional resistance determinants conferring resistance to other antimicrobial classes, including fluoroquinolones, trimethoprim-sulfamethoxazole, and aminoglycosides	*E. coli* *K. pneumoniae* *Klebsiella oxytoca* *Proteus mirabilis*	Routine ESBL testing is not performed by most clinical microbiology laboratories as current CLSI and EUCAST breakpoints should detect most ESBL isolates Non-susceptibility to ceftriaxone (i.e., ceftriaxone minimal inhibitory concentration ≥2 µg/ml) is often used as proxy for ESBL production^b^ Testing for ESBL confirmation and characterization may be warranted for infection control or epidemiologic purposes Molecular detection of ESBL genes can be obtained via commercially available systems capable of detecting genus/species targets, including *bla*_CTX-M_ gene	Worldwide
Carbapenemases						
*K. pneumoniae* carbapenemases (KPC)	Class A (serine)		Global spread of CRE has been facilitated by mobile genetic elements harboring genes encoding for carbapenemases Carbapenem-resistant organisms often carry additional plasmid-borne genes against other antimicrobial classes	*K. pneumoniae* *Enterobacter* spp. *E. coli* *K. oxytoca* *Serratia marcescens* *Citrobacter freundii*	Use current CLSI or EUCAST breakpoints for carbapenems to identify nonsusceptibility to imipenem, meropenem, doripenem, or ertapenem Knowledge of whether a CRE isolate is carbapenemase-producing and the specific carbapenemase is important in guiding clinical treatment decisions Phenotypic tests such as modified carbapenem inactivation method^c^ or Carba NP test^d^ differentiate carbapenemase and non-carbapenemase-producing CRE Molecular testing that can identify specific carbapenemase gene families is available. Carbapenemase phenotypic and/or genotypic testing is performed by certain clinical microbiology laboratories	Worldwide, particularly in the US, South American, southern Europe, Israel, and China
Metallo-β-lactamases, including NDM, VIM, IMP	Class B (zinc)			*K. pneumoniae* *E. coli* *K. oxytoca* *S. marcescens* *Enterobacter* *C. freundii*		NDM-1: India, Pakistan, China, northern Europe, Balkan countries VIM: southern Europe IMP: Japan, Southeast Asia
Oxacillinases (OXA-48-like)	Class D (serine)	Some remain susceptible to cephalosporins while resistant to carbapenems May be found on chromosome		*Acinetobacter baumannii* *Pseudomonas aeruginosa* *E. coli* *K. pneumoniae* *P. mirabilis* *C. freundii*		OXA-48-like: Turkey, Mediterranean basin, Middle East, northern Africa, India

CLSI, Clinical and Laboratory Standards Institute; EUCAST, European Committee on Antimicrobial Susceptibility Testing; US, United States; NDM, New Delhi metallo-β-lactamase; VIM, verona integron-encoded metallo-β-lactamase; IMP, imipenem hydrolyzing metallo-β-lactamase.

^a^ The Ambler classification is a classification system for β-lactamases based on the molecular structure (amino acid sequence and the active site residue). Classes A, C, and D have serine in the active site, whereas Class B uses zinc.

^b^ This threshold has limitations with specificity as organisms not susceptible to ceftriaxone for reasons other than ESBL production may be falsely presumed to be ESBL-producers. Other indicator cephalosporins that suggest likely ESBL production include cefotaxime, ceftazidime, cefpodoxime.

^c^ The carbapenem inactivation method (CIM) and modified CIM (mCIM) are phenotypic screening tests for qualitative detection of carbapenemase enzymes based on zone of inhibition results after incubation of a meropenem disk incubated in suspension of organism.

^d^ The Carba NP test is a phenotypic (colorimetric) biochemical test for the qualitative detection of carbapenemase enzymes in *Enterobacterales* and *Pseudomonas aeruginosa* based on *in vitro* hydrolysis of imipenem by a bacterial lysate. Results in 30 min to 2 h.

Although this review focuses on β-lactamases, antibiotic resistance in Gram negative organisms is complex. Other mechanisms can also be present individually or simultaneously with β-lactamases to create various resistance patterns. This can be illustrated with an important distinction in the heterogenous mechanisms of CRE. Carbapenem resistance may develop via two mechanisms: (1) production of a carbapenemase encoded by genes usually found on transmissible mobile elements (carbapenemase-producing CRE, CP-CRE) or (2) production of ESBL or AmpC β-lactamases combined with impaired membrane permeability from certain porin mutations or efflux pumps (termed non-CP-CRE). The Centers for Disease Control (CDC) definition of CRE represents both mechanisms in the CRE terminology, including members of the *Enterobacterales* order resistant to at least one carbapenem antibiotic or those producing a carbapenemase enzyme (9).

### 3.1. Incidence of MDRGN infections, including pediatric SOT and HCT

While the incidence of MDRGN infections reported among adult SOT and HCT recipients varies by geography and institution, overall rates of infection have steadily increased in recent years, with estimates showing an almost ten-fold rise in incidence between the early 2000's and 2015 (6). ESBL-producing *Enterobacterales* infections are the most common cause of MDRGN infections among SOT recipients and account for up to 75% of these infections (10–12). The incidence of CRE infection has ranged from 3% to 10% in areas of endemicity and has been best described in abdominal SOT recipients. Adult data also suggests increased mortality among transplant recipients with MDRGN infections, with 30-day mortality ranging from 14% to 26% with ESBL infection and 18%–41% in CRE infection (10–14).

Yet despite the increasing attention on MDR infections among adult transplant recipients, knowledge of the epidemiology of these infections in children remains limited. MDR infections have been increasingly identified in children and adolescents, although data, particularly in immunocompromised children, requires further study (1–4). While the prevalence of CRE infections in US children remains low overall, with the Surveillance Network (TSN) database-USA estimating 0.08% CRE vs. 0.47% ESBL in available isolates from children, rates continue to increase (4). Such infections have been associated with poor outcomes such as ICU admission and death in children (1, 2).

Research directly examining MDRGN infections in pediatric SOT or HCT recipients is scarce, but available data suggests the epidemiology, risk factors, and outcomes in the pediatric population may reflect trends observed among adults, with increased rates of MDRGN infection observed in the pediatric immunocompromised and transplant population. Among pediatric liver transplant recipients, the estimated incidence of MDR in a single-center retrospective study of 118 children in Thailand between 2010 and 2018 found 62.4% (58/93) of culture-proven bacterial infections post-transplant were due to MDRGN isolates, with a predominance of *Klebsiella pneumoniae* and *Escherichia coli* (15). These were largely intra-abdominal infections, accounting for 47.9% of infections. Another recent single-center retrospective study of pediatric liver transplant recipients from the United Kingdom found a lower rate, noting 27% of patients had colonization with MDRGN with an infection rate of 16.6% (16). MDR organisms have also been identified as a significant cause of severe sepsis for the pediatric liver transplant population as well, accounting for 47.6% (20/42) of organisms causing bacterial sepsis in a tertiary pediatric intensive care unit in the United States. It should be noted though that MDR organisms in this study were defined by resistance to one or more classes of antibiotics and also included cases of vancomycin resistant *Enterococcus* (accounting for 9 of the 20 episodes of MDR organisms) (17). Similar to adult studies, ESBL infections accounted for the majority of MDR Gram-negative infections in these pediatric liver transplant recipients, and notably, CRE infection rates were found to range from 3% to 17% (15–17).

Knowledge of the incidence and outcomes of MDRGN infections in children receiving treatment for malignancy or after HCT is also limited. In a multi-national, multicenter retrospective study of 1,291 bloodstream infections in pediatric patients with allogeneic HCT or those treated with chemotherapy, more than 25% of Gram-negative infections were resistant to ceftazidime, cefepime, piperacillin-tazobactam, and ciprofloxacin. CRE represented 9% of infections. This study found that bloodstream infection with MDRGN infection was significantly associated with ICU admission and death (18).

### 3.2. Risk factors for MDRGN infections

Risk factors for MDR infections in immunocompromised adults have been well described and include prior exposure to antimicrobial drugs, critical illness, prolonged and frequent exposure to health care, residence in long term facilities, pre-transplant colonization, and longer length of stay prior to infection (11). In children, previous exposure to antibiotics, particularly third generation cephalosporins and carbapenems, has been significantly associated with MDRGN bloodstream infection and post-transplant infection in pediatric patients with hematologic malignancy, HCT, and liver transplantation (15, 18). Other noted factors in Phichaphop, et al. included operation duration and length of ICU stay (15).

The molecular and geographic epidemiology of β-lactamase producing organisms, particularly carbapenemases, is an important consideration when approaching diagnostic and empiric treatment strategies. In the United States, the *K. pneumoniae* carbapenemase (KPC) enzyme is the predominant carbapenemase found in CP-CRE isolates infecting adults and children, while other carbapenemases are infrequently identified (8, 19–21). The class of metallo-β-lactamase (MBL) carbapenemases are unique as they are not inhibited by avibactam, vaborbactam, or relebactam. Given this has a significant impact on the available treatment options, clinicians must utilize caution and have clinical suspicion if carbapenem resistance is detected in isolates from children with epidemiologic links to MBL-endemic regions. Additionally, sporadic domestic acquisition of other MBLs remain possible (8, 22, 23).

## 4. General diagnostic and evaluation strategies

### 4.1. Approach to initial evaluation

Infection due to a MDRGN organism should be suspected in an immunocompromised or transplant recipient patient when there is a lack of clinical response to antimicrobial therapy, risk factors present for possible MDR organism exposure, and/or previous isolation or colonization of such pathogen. Early involvement of pediatric transplant ID specialists in these complex cases is important to assist with additional clinical evaluation, which should be guided by the patient's presentation, signs, and symptoms. Aggressive evaluation should be pursued to identify the source of infection, and relevant imaging studies may be necessary. Sampling with bacterial cultures from blood as well as any other suspected sites of infection should be obtained as clinically feasible to guide therapy. Source control, including drainage of infected fluid collections or removal of infected devices or hardware, are important mainstays of therapy, especially given the limited antimicrobial options for MDR Gram-negative infections.

### 4.2. Evaluating for antimicrobial resistance

If a clinical isolate is available, the first step in evaluation of antimicrobial resistance is examining the AST panel results to assess for the possible mechanism(s) of resistance via implementation of interpretive criteria from the Clinical & Laboratory Standards Institute (CLSI) or European Committee on Antimicrobial Susceptibility Testing (EUCAST) (24, 25). Often additional testing may be necessary (such as requesting additional local or send-out susceptibilities to novel agents), and isolates should be evaluated in laboratories experienced with recognition of complex resistance mechanisms.

Ceftriaxone-resistance is used as a common proxy to identify ESBL-producing bacteria. Routine ESBL testing is not performed by most clinical microbiology laboratories as current CLSI and EUCAST breakpoints should detect most ESBL isolates, but testing for ESBL confirmation and characterization may be warranted for infection control or epidemiologic purposes.

For identification of CRE, the current CLSI or EUCAST breakpoints are used to identify nonsusceptibility to imipenem, meropenem, doripenem, or ertapenem. *Enterobacterales* that are resistant to at least one of the carbapenems listed are considered CRE based on the CDC definition, but this definition could include both CP-CRE and non-CP-CRE as noted previously (9). Both CLSI and EUCAST define *Enterobacterales* susceptibility to ertapenem and doripenem the same (MICs of ≤0.5 μg/ml and ≤1 μg/ml, respectively), but there is some variation in the breakpoints for *Enterobacterales* and meropenem/imipenem. CLSI defines susceptibility to meropenem and imipenem with MICs of ≤1 μg/ml, while meropenem and imipenem MICs ≤2 μg/ml are considered susceptible by EUCAST (24, 25).

Although the presence of carbapenemase does not influence the categorization of susceptibility, carbapenemase detection and characterization may be recommended for public health and infection control purposes. Additionally, knowledge of whether a CRE isolate is carbapenemase-producing and the specific carbapenemase is also important to guide clinical decisions (which will be discussed further in the following treatment section). Lab tests are available to identify the presence or absence of a carbapenemase, including the Carba NP assay or the modified carbapenem inactivation method (mCIM). Molecular tests can be used to identify specific carbapenemase genes. It is important to note that “negative” results on molecular carbapenemase testing should not be interpreted as confirmation of the absence of carbapenemase (as other carbapenemase genes may be present that are not included in the assay used) or carbapenem susceptibility (as the isolate may be a non-CP CRE). The CLSI does recommend use of the Carba NP assay, mCIM, and/or a molecular assay when *Enterobacterales* isolates have an imipenem or meropenem MIC of 2–4 μg/ml or ertapenem MIC of 2 μg/ml.

The recommended diagnostic approach for identification of ESBL and carbapenemases are the focus of this review and are summarized in Table 1. A few other notable MDRGN organisms include *Pseudomonas aeruginosa* with difficult-to-treat resistance (DTR-*P. aeruginosa*; defined as non-susceptibility to piperacillin-tazobactam, ceftazidime, cefepime, aztreonam, meropenem, imipenem-cilastatin, ciprofloxacin, and levofloxacin; illustrated in Case 2), carbapenem-resistant *Acinetobacter baumannii* (CRAB), and *Stenotrophomonas maltophilia*. CLSI and EUCAST interpretative criteria for antimicrobial susceptibilities are also utilized for these organisms. It is useful to note that carbapenemase production is a rare cause of carbapenem resistance in *P. aeruginosa* isolates in the US and generally carbapenemase testing of clinical isolates of *P. aeruginosa* is not as critical as for CRE clinical isolates in US hospitals. That said, carbapenemases have been identified in up to one-fifth of carbapenem-resistant *P. aeruginosa* in other regions of the world and current IDSA guidance encourages performing additional susceptibility testing for novel β-lactam agents (7).

## 5. Treatment strategies

The treatment of MDRGN infections in the SOT and HCT recipient requires a high index of clinical suspicion with an appreciation of the unique anatomic, immunologic, and environmental risks predisposing individuals to infection. Appropriate empiric antibiotic therapy, coupled with adequate source control and durations tailored to the nature of infection remain the cornerstone of therapy. Among transplant recipients, such consideration should take into account impaired or absent neutrophil function, the risk of anastomotic leakage, and the frequent presence of central venous catheters, foley, peritoneal drains, and hemodialysis catheters (26).

Available antimicrobial treatment regimens for MDRGN infections have rapidly evolved in the past decade, particularly with the addition of novel β-lactam-β-lactamase inhibitor (BLBLI) agents. The optimal treatment of these infections remains complex though given resistance mechanisms and a limited armamentarium, particularly with CRE organisms. Antimicrobial management in children is further complicated by limited pediatric-specific pharmacokinetic and pharmacodynamic data and few clinical trials evaluating novel agents for use in the pediatric population.

### 5.1. Selecting empiric therapy

Selection of an appropriate empiric antibiotic regimen is essential to reduce morbidity and mortality risk following transplantation, with inappropriate empiric antimicrobial regimens associated with up to a three-fold increased mortality risk in adult HCT recipients (27). Factors that should be considered in choosing an empiric antibiotic regimen include local resistance patterns (particularly ESBL and regional carbapenemases prevalence), prior patient history of infection or colonization, recent antimicrobial exposure, and severity of illness. Empiric antimicrobial regimens following transplantation should demonstrate robust activity against *Pseudomonas aeruginosa*. Carbapenem agents, such as meropenem or imipenem, should be considered in settings of high local ESBL prevalence, known prior history of ESBL infection or colonization, and/or critical illness. The empiric use of novel BLBLIs (such as ceftazidime-avibactam, ceftolozane-tazobactam, imipenem-cilastatin-relebactam) or other novel agents is generally avoided if possible but should be considered in the setting of known or suspected risk factors for CRE.

### 5.2. ESBL producing *Enterobacterales* (ESBL-E)

For bloodstream and other serious non-urinary tract infections caused by ESBL-E, treatment with carbapenem therapy should be utilized, with meropenem and imipenem preferred over ertapenem in cases of critical illness. Newer BLBLIs and cefiderocol are active against ESBL-producing organisms but are not utilized as first line if other options are available to preferentially reserve these agents for carbapenem resistant organisms.

### 5.3. CRE

The results of carbapenem susceptibility testing should guide treatment selection, although antibiotic choice should generally not be stratified based on the presence of carbapenemase production (CP-CRE vs. non-CP-CRE). To start, carbapenem susceptibility testing can initially provide guidance on whether an extended infusion carbapenem can be used vs. a newer BLBLI (22). For isolates that exhibit susceptibility to meropenem and imipenem but are not susceptible to ertapenem, the use of extended-infusion meropenem (or imipenem-cilastatin) is suggested, assuming no carbapenemase has been identified. Extended-infusion β-lactam (typically over 3 h) optimizes killing time and increases the likelihood of achieving target drug levels. This approach is often used in settings of high inoculum or refractory infection as well.

If the CRE isolate has a meropenem MIC ≥4 µg/ml (such as in Case 1), the use of a novel BLBLI (ceftazidime-avibactam, meropenem-vaborbactam, or imipenem-cilastatin-relebactam) is preferred. If specific carbapenemase testing is available and identifies a specific carbapenemase, this can further inform the selection of the optimal BLBLI given differential activity based on carbapenemase type. For example, while these three agents all have a high likelihood of activity against KPC-producing organisms as well as non-CP-CRE, meropenem-vaborbactam and imipenem-cilastatin-relebactam do not retain activity against OXA-48-like carbapenemases. MBL-producing organisms present further challenges in treatment as neither ceftazidime-avibactam, meropenem-vaborbactam, nor imipenem-cilastatin-relebactam have activity.

For patients with suspected or confirmed infection due to MBL-producing organisms or with previous clinical or surveillance cultures for MBL, preferred treatment options include the combination of ceftazidime-avibactam plus aztreonam (used in conjunction to create “aztreonam-avibactam”) or cefiderocol monotherapy. MBLs, such as NDM, hydrolyze penicillins, cephalosporins, and carbapenems—but not aztreonam. Aztreonam is active against MBLs but may not be effective as monotherapy due to co-production of ESBLs, AmpC β-lactamases, or other carbapenemases such as KPC or OXA-48-like. Avibactam does not have an effect on MBL itself but can “protect” aztreonam from hydrolysis due to ability to inhibit these β-lactamases co-produced by most MBL isolates. Of note, avibactam restores activity of aztreonam in MBL-producing *Enterobacterales*, but not MBL-producing *P. aeruginosa* due to additional non-β-lactam mechanisms of resistance in *P. aeruginosa* (28). Specific synergy antimicrobial susceptibility testing of this combination therapy is available in very limited clinical labs, but no specific lab method is endorsed by CLSI. There is clinical experience with combination of ceftazidime-avibactam plus aztreonam but it is not FDA-approved and requires careful attention to the logistics of co-administration (29, 30). Another MBL-producing CRE option is cefiderocol, which exhibits activity against any of the major carbapenemase enzymes as well as non-CP-CRE. Generally cefiderocol is preserved for cases where other β-lactams cannot be used, such as MBL.

If carbapenemase testing is not available or is negative, novel BLBLIs remain the preferred treatment option, unless there is a possible epidemiologic link/exposure to MBL-producing organism or prior clinical or surveillance cultures with an MBL-producing organism.

Other agents to note for management of CRE include aminoglycosides, polymyxins (such as polymyxin B or colistin), plazomicin, tigecycline, eravacycline, and omadacycline. Aminoglycosides and polymyxins were historically the backbone for treatment of CRE, but the newer BLBLIs are favored now due to clinical success, less complex dosing regimens, and less risk of nephrotoxicity. Aminoglycoside monotherapy is generally reserved for urinary tract infections. Tigecycline and eravacycline are alternative options for CRE infections not involving the bloodstream or urinary tract and their activity is independent of type of carbapenemase.

### 5.4. MDR- and DTR-*P. aeruginosa*

MDR-*P. aeruginosa* isolates demonstrating carbapenem resistance but susceptibility to traditional anti-pseudomonal agents can usually be treated effectively with traditional beta-lactams via extended infusion. In cases of critical illness or poor source control, novel agents such as ceftolozane-tazobactam, ceftazidime-avibactam, and imipenem-cilastatin-relebactam are viable alternatives, although this is not the otherwise preferred option to preserve effectiveness of these agents for future use in increasingly antibiotic-resistant infections.

DTR-*P. aeruginosa* should be treated with ceftolozane-tazobactam, ceftazidime-avibactam, or imipenem-cilastatin-relebactam. Ceftolozane and ceftazidime have a similar structure, but a benefit of ceftolozane is the decreased impact by hydrolysis and porin loss compared to ceftazidime. Ceftolozane has independent anti-pseudomonal activity that does not rely on tazobactam and the combination of ceftolozane-tazobactam is relatively stable to all pseudomonal β-lactam resistance mechanisms. The patient in Case 2 was transitioned to ceftolozane-tazobactam for definitive therapy.

Cefiderocol is an alternative treatment option that is reserved for when there is inactivity, intolerance, or unavailability of the newer BLBLIs. In the case of infection with a DTR-*P. aeruginosa* isolate that is MBL-producing though, cefiderocol would be the drug of choice.

### 5.5. Use of combination therapy

The possible benefit of combination therapy with multiple agents has been explored, but ongoing use once susceptibility testing is available is controversial. Empiric combination antimicrobial therapy, such as use of β-lactams or BLBLIs with aminoglycosides or possibly polymyxins, is often deployed initially to increase the likelihood that at least one agent administered is active against infection in patients at high risk for MDRGN infections, especially CRE and MDR/DTR-*P. aeruginosa*. However, once a β-lactam agent demonstrates activity, there is no clear established benefit of continued combination therapy over monotherapy in CRE or DTR-*P. aeruginosa* infections. There is no randomized trial data comparing monotherapy vs. combination therapy, but the current IDSA panel guidance suggests against the use of combination therapy in these cases of CRE or DTR-*P. aeruginosa* infection given the lack of clear benefit in available studies and risk of antibiotic associated adverse events with multiple antibiotics (7).

In contrast, combination therapy is suggested for infections due to CRAB and MDR *Stenotrophomonas maltophilia*. While the incidence of CRAB infection remains low among pediatric transplant recipients, the general approach for treatment would include use of high-dose ampicillin-sulbactam (regardless of susceptibility results) in combination with at least one other active agent, given the limited clinical data supporting any single antibiotic. The production of OXA carbapenemases leads to resistance to β-lactams in most CRAB isolates, but these isolates may also produce MBLs and other serine carbapenemases. Sulbactam-durlobactam is a recently FDA-approved agent with activity against CRAB. Durlobactam inhibits class A, C, and D β-lactamase enzymes commonly produced by CRAB, restoring activity of sulbactam. Specific pediatric data on this agent is not available at this time. *S. maltophilia* infections pose similar challenges to CRAB infections, and treatment selection is hindered by resistance often caused by MBL and other β-lactamases, intrinsic resistance, and accumulation of efflux pumps. These infections are usually addressed with trimethoprim-sulfamethoxazole in combination with another agent. Another possibility is use of the ceftazidime-avibactam and aztreonam combination in cases of critical illness, intolerance, or inactivity of other agents.

### 5.6. Definitive therapy and duration of therapy

A summary of the available antimicrobial agents and general treatment recommendations by organism are also described in Tables 2, 3. Ultimately the final antibiotic regimen will require consideration of susceptibility results, ability to obtain adequate drug levels at site of infection, side effect profile, route of administration, and cost/formulary considerations. Children in need of transplantation may have chronic and prolonged bacterial infections in addition to their underlying organ dysfunction and remain at risk for recurrent and relapsing infections following transplantation (9). As such, in addition to effective antimicrobial regimens, adequate source control, awareness of post-transplant co-morbidities, and consideration of overall host immune defenses are critical in efforts to control bacterial infection following SOT/HCT. Standard recommended antimicrobial durations in SOT and HCT recipients are often confounded by impaired immunologic clearance, high risk of recurrence, and persisting sources of infection, and as such, durations such should be guided by interval clinical improvement, restoration of immune function and demonstrated source control.

**Table 2:** Summary and activity of FDA-approved antibiotics against multidrug-resistant organisms^a^.

Drug	Class/ mechanism	Year of FDA approval (year of pediatric approval if granted)	Expected activity							
			ESBL	CRE				MDR /DTR- *Pseudo monas aerugin osa*	CRAB	*Stenotr ophom onas maltop hilia*
				KPC	MBL	OXA-48-like	Non-CP			
Ceftolozane-tazobactam	BLBLI	2014 (2022)								
Ceftazidime-avibactam	BLBLI	2015 (2019)								
Meropenem-vaborbactam	BLBLI	2017								
Imipenem-cilastatin- relebactam	BLBLI	2020								
Cefiderocol	Cephalosporin	2019								
Plazomicin	Aminoglycoside	2018								
Eravacycline	Tetracycline	2018								
Omadacycline	Tetracycline	2018								
Sulbactam-durlobactam	β-lactamase inhibitor	2023								

FDA, United States Food and Drug Administration; BLBLI, β-lactam-β-lactamase inhibitor; ESBL, extended spectrum β-lactamase; CRE, carbapenem resistant *Enterobacterales*; KPC, *Klebsiella pneumoniae* carbapenemase; MBL, metallo-β-lactamase; OXA, oxacillin carbapenemase; non-CP, non-carbapenemase-producing *Enterobacterales*; CRAB, carbapenem-resistant *Acinetobacter baumannii*.

^a^ This table represents a guide, but ultimately the choice of antimicrobial should be guided by the available susceptibility testing results and current CLSI/EUCAST breakpoints.

**Table 3:** Summary of treatment recommendations for systemic or severe MDR gram negative infections^a^.

Organism	Recommended management options
All	• Source control is paramount • Early involvement and consultation with Transplant Infectious Diseases specialists as well as physician or pharmacist members of local antibiotic stewardship program • Ensure appropriate infection control and prevention measures
ESBL-producing *Enterobacterales*	• Carbapenems are drugs of choice (meropenem, imipenem-cilastatin, ertapenem)^b^ • Meropenem or imipenem-cilastatin preferred if critically ill^c^ • Do not use piperacillin-tazobactam or cefepime, even if susceptibility is demonstrated
CRE	• For *Enterobacterales* isolates that exhibit susceptibility to meropenem and imipenem (MICs ≤1 µg/ml) but not susceptible to ertapenem (MIC ≥1 µg/ml): ◦ Extended infusion meropenem (or imipenem-cilastatin) • If carbapenemase testing results are not available or negative: ◦ Ceftazidime-avibactam ◦ Meropenem-vaborbactam ◦ Imipenem-cilastatin-relebactam • If confirmed KPC-producing infection: ◦ Ceftazidime-avibactam ◦ Meropenem-vaborbactam ◦ Imipenem-cilastatin-relebactam • If epidemiologic risk for MBL-producing organism or previous clinical or surveillance culture with MBL-producing isolate: ◦ Cefiderocol monotherapy ◦ Combination of ceftazidime-avibactam + aztreonam • If OXA-48-like-producing infection: ◦ Ceftazidime-avibactam (preferred) ◦ Cefiderocol (alternative) • Alternative options when β-lactams are not active or unable to be tolerated: ◦ Cefiderocol ◦ Tigecycline ◦ Eravacycline
MDR/DTR-*Pseudomonas aeruginosa*	• If isolate susceptible to traditional non-carbapenem β-lactam agent (such as piperacillin-tazobactam, ceftazidime, cefepime, aztreonam) and carbapenems, the former are preferred • If isolate non-susceptible to any carbapenem agent but susceptible to other β-lactam agent, utilize the traditional β-lactam with high dose extended-infusion therapy ◦ If critically ill or poor source control, could consider use of ceftolozane-tazobactam, ceftazidime avibactam, imipenem-cilastatin-relebactam • If DTR-*P. aeruginosa*: ◦ Ceftolozane-tazobactam ◦ Ceftazidime-avibactam ◦ Imipenem-cilastatin-relebactam ◦ Alternative: cefiderocol ◦ If MBL, preferred treatment is cefiderocol
Carbapenem-resistant *Acinetobacter baumannii* (CRAB)	• High dose ampicillin-sulbactam in combination with at least one other active agent (such as polymyxin B, high dose minocycline, high dose tigecycline) • Cefiderocol should be limited to refractory cases
*Stenotrophomonas maltophilia*	• Use of two active agents: trimethoprim-sulfamethoxazole, minocycline/tigecycline, cefiderocol, or levofloxacin • Can also utilize combination of ceftazidime-avibactam + aztreonam

MDR, multidrug resistant; ESBL, extended spectrum β-lactamase; CRE, carbapenem-resistant *Enterobacterales*; DTR, difficult-to-treat resistance; MBL, metallo-β-lactamase.

^a^ These recommendations are consistent with the IDSA 2023 Guidance on the Treatment of Antimicrobial Resistant Gram-Negative Infections, which were published 6/7/2023 (7). Recommendations focus on the preferred antibiotic choices and alternatives for treatment of serious MDRGN infections outside of the urinary tract.

^b^ This recommendation is based on a randomized clinical trial where higher mortality was described in adults with ceftriaxone non-susceptible *E. coli* and *K. pneumoniae* bloodstream infection (87% with ESBL genes) who were treated with piperacillin-tazobactam vs. meropenem (7, 31).

^c^ Ertapenem is highly protein bound compared to meropenem and imipenem, leading to a relatively prolonged serum half-life. In critical illness, the increased free fraction of ertapenem will lead to a decrease in serum half-life.

### 5.7. Patient case follow-up

The patient in Case 1 required several prolonged courses of antibiotics which would improve his symptoms temporarily, so he was ultimately listed for transplant as treatment for recurrent cholangitis. He underwent successful deceased donor liver transplant 1 month later with subsequent resolution of his infections. The patient in Case 2 underwent successful skin graft procedure on day 21 of illness, and ceftolozane-tazobactam was continued for an additional week post-debridement with subsequent clinical resolution and wound healing.

## 6. Preventive strategies

Prevention of MDR gram-negative infections in transplant candidates and recipients requires consideration of both intrinsic, patient-specific factors, as well as broader hospital-level infection control measures.

### 6.1. Patient-specific factors

Patient-specific factors include prolonged hospitalization in the pre- and peri-transplant setting due to poor functional status or complications related to the underlying diagnosis, indwelling medical devices such as central venous catheters and ventricular-assist devices, and repeated antibiotic treatment courses (11). Such factors may lead to selection for resistant organisms and persistent colonization with these organisms, which may drive increasingly broad antibiotic selection in the setting of fevers and other signs of infection. Antimicrobial stewardship interventions to optimize antibiotic use in both the pre- and post-transplant setting are essential, and the lack of antibiotic de-escalation and use of expansive broad-spectrum empiric antibiotics for prolonged durations represent opportunities for improvement in transplant patients (11). In addition, efforts to remove indwelling medical devices as soon as clinically feasible can reduce risk of infection. Improving the functional status of patients, addressing nutrition and growth needs, and timely progression to transplant to alleviate complications associated with a failing organ or attain immunologic recovery are also critical for preventing infections, but many competing factors can influence the ability to achieve these goals.

### 6.2. Hospital-level infection control measures

Hospital-level infection control and prevention practices can ensure that MDR gram-negative infections are not transmitted within hospital settings. The Centers for Disease Control (CDC) developed a toolkit for healthcare facilities on the prevention of transmission of MDR organisms, specifically for CRE (summarized in Table 4), and the Cystic Fibrosis Foundation Infection Prevention and Control Guidelines should be implemented where transplant recipients with cystic fibrosis access care (32, 33). The benefit of routine surveillance of all patients with MDR organisms, including CRE, in healthcare settings or even on transplant floors is unclear. Only a small proportion of patients colonized with MDR organisms go on to develop clinically significant infections, and optimal infection control measures for transplant candidates or recipients known to be colonized with MDR organisms is uncertain. There is limited evidence of support surveillance outside of the outbreak setting, and intestinal decolonization for patients with known ESBL and CRE carriage is not recommended (11).

**Table 4:** General infection prevention and control strategies to reduce transmission and acquisition of MDRGN pathogens, such as CRE, in healthcare facilities (32, 33).

Surveillance	• Healthcare facilities should be aware of whether CRE has been isolated from patients admitted to their facility as well as whether their laboratories have capacity to perform CRE screening and carbapenemase testing • Benefit of routine surveillance of all patients with MDR organisms is unclear, but facilities should consider ongoing evaluation to quantify the incidence of CRE organisms
Hand hygiene	• Promote hand hygiene • Monitor adherence and provide feedback • Ensure access to hand hygiene stations
Contact precautions (CP)	• Generally, CRE colonized or infected patients in acute care facilities should be placed on CP. In lower acuity settings, the use of CP should be guided by potential risk based on functional and clinical status • Empiric CP can be considered for patients transferring from high-risk settings, pending results of surveillance testing • Educate and train healthcare personnel about CP including opportunities for donning and doffing practice • Monitor CP adherence and provide feedback
Education	• Education about risk factors for routes of transmission and preventative strategies (with a focus on hand hygiene and contact precautions) should be provided to patient, families, and healthcare personnel
Minimize use of invasive devices	• Such as central venous catheters, endotracheal tubes, and urinary catheters • Device use should be reviewed regularly to ensure they are still required • Devices should be discontinued promptly when no longer needed
Timely notification from laboratory when MDR organisms are identified	
Communication of CRE status for infected and colonized patients at discharge and transfer	• Transferring facilities should notify receiving facility of the presence of infection or colonization with CRE
Promotion of antimicrobial stewardship	• Antimicrobial stewardship programs and facilities should work to ensure antimicrobial are used for appropriate indications and duration
Environmental cleaning	• Facilities should perform daily cleaning that includes areas in close proximity to patient to decrease burden of organisms as well as terminal cleaning of patient room after discharge
Patient and staff cohorting	• When available, cohort CRE colonized or infected patients and the staff that care for them even if patients are housed in single rooms • If the number of single patient rooms is limited, reserve these rooms for patients with highest risk of transmission (e.g., incontinence)
Screening contacts of CRE patients	• Screen patient with epidemiologic links to unrecognized CRE colonized or infected patients
Chlorhexidine bathing	• Bathe patients in 2% chlorhexidine

### 6.3. Targeted pre-emptive or prophylactic treatment

Targeted screening of known contacts of patients with MDR infections and active surveillance of patients who meet specific risk criteria for MDR infections may be a more useful approach, particularly to identify the need for contact precautions and patient cohorting (33). For transplant candidates and recipients with known colonization with MDR organisms, pre-emptive treatment or ongoing prophylaxis, in the absence of evidence of clinical infection, is not recommended. Such use of prophylaxis has not demonstrated benefit for clearing colonization and raises concern for development of additional resistance through selective pressure.

Transplant candidates with known colonization with or history of MDR infections, including CRE, should not be excluded from transplantation based on this feature alone, with rare exceptions. SOT donors with MDR infection or colonization should also not be excluded, but communication between donor and recipient healthcare facilities is essential. If a donor is bacteremic or if there is evidence of infection of the allograft being transplanted, recipients will require appropriate post-transplant antibiotic treatment based on susceptibility testing of the identified organism.

## 7. Conclusions

Pediatric transplant ID specialists must remain vigilant to ensure rapid evaluation and management for MDR Gram-negative infections in pediatric transplant recipients. Despite limited data in the pediatric populations, risk factors described in these studies and prior adult experiences point to the potential consequences in vulnerable post-transplant recipients exposed to broad-spectrum antibiotics, frequent hospitalizations, and need for invasive procedures and indwelling devices. The approach to MDRGN infections among children following transplantation ultimately requires the optimization of preventive strategies, in conjunction with a high index of suspicion, effective antimicrobial strategies and source identification and control.
